# Weekend admissions and mortality for major acute disorders across England and Wales: record linkage cohort studies

**DOI:** 10.1186/s12913-019-4286-8

**Published:** 2019-09-02

**Authors:** Stephen E. Roberts, Ann John, Keir E. Lewis, Jonathan Brown, Ronan A. Lyons, John G. Williams

**Affiliations:** 1 Swansea University Medical School, Swansea University, Singleton Park, Swansea, UK; 2 Health Data Research UK, Swansea University, Swansea, UK; 3 Department of Respiratory Medicine, Prince Philip Hospital, Llanelli, UK; 4 Department of Gastroenterology, Gloucestershire Royal Hospital, Gloucester, UK

**Keywords:** Mortality, Weekend admissions, Acute disorders

## Abstract

**Background:**

To establish which major disorders are susceptible to increased mortality following acute admissions on weekends, compared with week days, and how this may be explained.

**Methods:**

Cohorts based on national administrative inpatient and mortality data for 14,168,443 hospitalised patients in England and 913,068 in Wales who were admitted for 66 disorders that were associated with at least 200 deaths within 30 days of acute admission. The main outcome measure was the weekend mortality effect (defined as the conventional mortality odds ratio for admissions on weekends compared with week days).

**Results:**

There were large, statistically significant weekend mortality effects (> 20%) in England for 22 of the 66 conditions and in both countries for 14. These 14 were 4 of 13 cancers (oesophageal, colorectal, lung and lymphomas); 4 of 13 circulatory disorders (angina, abdominal aortic aneurysm, peripheral vascular disease and arterial embolism & thrombosis); one of 8 respiratory disorders (pleural effusion); 2 of 12 gastrointestinal disorders (alcoholic and other liver disease); 2 of 3 ageing-related disorders (Alzheimer’s disease and dementia); none of 7 trauma conditions; and one of 10 other disorders (acute renal failure). Across the disorders, 64% of the variation in weekend mortality effects in England and Wales was explained by reductions in admission rates at weekends and the medical disease category.

**Conclusions:**

The effect of weekend admission on 30 day mortality is seen mainly for cancers, some circulatory disorders, liver disease and a few other conditions which are mainly ageing- or cancer-related. Most of the increased mortality is associated with reduced admission rates at weekends and the medical disease category.

**Electronic supplementary material:**

The online version of this article (10.1186/s12913-019-4286-8) contains supplementary material, which is available to authorized users.

## Background

A so-called ‘weekend mortality effect’ of increased mortality for hospital admissions on weekends - compared with normal week days - has been the subject of increased interest and controversy in recent years. [1–3] However, explanations for the variable evidence reported from numerous studies, often based on differing settings, methodologies and medical conditions, is still lacking.

Some studies that have reported on weekend mortality effects investigated single disorders, such as stroke, [4–8] acute myocardial infarction, [9–12] or hip fracture, [13–15] or individual services such as dedicated stroke units, [7, 8] specialist trauma centres, [14, 16–18] kidney transplantation, [19] and primary percutaneous coronary intervention services. [9–11] These were mostly based in single specialist centres, networked or audited centres and usually reported little or no weekend mortality effects. Other studies have focused on all hospital admissions, [1, 2, 20] or particular types of admission, aggregated nationally or regionally, such as elective surgery, [21] emergency surgery, [18, 22, 23], acute unselected admissions, [24–31] or have reviewed all types of condition, [32] and have mostly reported strong overall evidence of weekend effects. Several studies have reported on weekend effects for a range of disorders. [24, 31, 33, 34]

A recent review on the future direction of research into the weekend effect has highlighted the need for more evidence from large population studies that investigate and analyse individual diseases. [35] The primary objective of this study was to focus on all major acute disorders and to establish which are susceptible to weekend mortality effects following acute admissions. Further objectives were to test if weekend mortality effects could be explained by factors including reductions in admission rates at weekends, the type of medical condition, mortality risk soon after admission, changes in the source of admission and patient socio-demographics. The rationale behind these explanatory factors includes, firstly, greatly reduced admissions and increases in the proportion of admissions through emergency departments at weekends may reflect higher condition severity thresholds for admission at weekends. Secondly, weekly variation in admissions with high mortality risk soon after admission and for different types of medical condition may be linked to the availability of some specialist services at weekends.

To strengthen the evidence, for confirmatory purposes, the study was designed using data collected independently from two different national health services across England and across Wales. The main a priori hypothesis was that weekend effects would be largest for specific types of medical condition that have greatly reduced admission rates at weekends or particularly high mortality during acute phases.

## Methods

### Study cohorts

The study used cohorts of adults (aged 18+ years) who were admitted unscheduled for major disorders to all public hospitals across England and Wales from January 1st 2004 to December 31st 2012. These cohorts were based on national administrative inpatient data, Hospital Episode Statistics for England (population 53.5 million in 2012) and the corresponding Patient Episode Database for Wales (population 3.07 million). The inpatient data were systematically linked to mortality data from the Office for National Statistics and the Welsh Demographic Service to identify all deaths that occurred following discharge from hospital, as well as inpatient deaths, within 30 days of admission. The information sources were compiled, stored and accessed through a secure, privacy protected, data storage gateway, the Secure Anonymised Information Linkage (SAIL) databank, [36] supported by The Farr Institute of Health Informatics Research. The ascertainment of mortality and the record linkage methodology, based on a unique anonymised, encrypted linking field for each patient, have been validated as > 98 and > 99.8% accurate. [37] We included each person’s first emergency admission following the start of the study period and then also included any subsequent admissions provided they occurred at least 30 days following discharge from a preceding emergency admission.

### Study disorders

The study cohorts included disorders that led to substantial mortality, which was defined as 200 deaths or more within 30 days of acute admission during the study period in both England and Wales. 66 disorders fulfilled these criteria. They were circulatory (13 conditions), respiratory (8), gastrointestinal (12), trauma (7), cancers (13), (other) ageing-related conditions (3) and various other disorders (10). The ageing-related conditions were defined by a mean patient age at admission > 80 years and were Alzheimer’s disease, dementia and age-related physical debility. See Appendix, Table 4 for a full list of the 66 study disorders and the corresponding ICD-10 codes used to define them.

### Outcome measures

The primary study outcome measure was the weekend mortality effect, which was defined as percentage increased or decreased mortality at 30 days following admissions on weekends compared with admissions on week days, based on odds ratios. Weekends were defined as 00:00 h on Saturday to 23:59 h on Sunday. Public holidays were not counted as weekend or week day admissions and were excluded from the analyses of weekend mortality effects. The secondary outcome measure was mortality at 30 days, calculated using the numbers of admissions for each disorder as denominators and the numbers of deaths (from all causes) as numerators.

### Exposure measures

Study exposure measures for each of the 66 study disorders were, first, the medical disease category (circulatory, respiratory, gastrointestinal, trauma, cancers, ageing-related and all other disorders) Second, reductions in admission rates at weekends, compared with week days. Third, the risk of mortality on the day of admission. Fourth, changes in the source of admission at weekends (ratio of admissions via hospital emergency departments compared with primary care and consultant clinics). Last, mean patient age and patient social deprivation. [38, 39] All exposure measures except medical disease category were grouped into quintiles with equal numbers of study disorders in each quintile (see Appendix, Table 5 for further details of these exposure measures and the quintile categories).

### Methods of analysis

For each of the 66 study disorders in both populations, multivariable logistic regression models were fitted individually to adjust weekend mortality effects (mortality odds ratios for weekend vs week day admissions), as the outcome measures, for patient age (in 15 quinquennial age groups from < 35, 35–39 up to 85+ years, with < 35 years as the reference category), sex and eleven major patient co-morbidities. As a sensitivity analysis, we adjusted for age as a continuous variable instead of the conventional quinquennial age groups, but this made little or no difference to the study findings. The co-morbidities were ischaemic heart disease (ICD-10 codes, 120-I25), other cardiovascular diseases (I00-I15, I26-I52), cerebrovascular disease (I60-I69), other circulatory diseases (I70-I99), malignancies (C00-C97), liver disease (K70-K77), COPD (J40-J44), asthma (J45, J46), diabetes (E10-E14), renal failure (N17-N19) and dementia (F00-F03, F05.1, G30). The co-morbidities were based on a diagnosis recorded anywhere in the current or in previous inpatient records during the preceding five years. In the multivariable modelling, to eliminate possible biases in the determination of patient co-morbidities from inpatient admissions alone, adjustment was also made for patients with no previous admissions during the preceding five years.

Statistical significance was measured at the conventional 5% level. However, with 66 study conditions, as significance is affected by multiple testing, to identify and comment on conditions with weekend effect sizes that are unlikely to be false positive findings, we arbitrarily defined a ‘large weekend mortality effect’ as a significant weekend effect size of > 20%. Nonetheless, weekend effect sizes of any magnitude are presented and assessed throughout the analysis. As statistical significance is also affected by the much larger population of England than Wales, we placed greater importance on the findings from England by assessing significant weekend effects, firstly, in the larger population of England and then, secondly, for additional confirmatory purposes in Wales.

In both populations, multivariable linear regression was used to assess the degree of variation in weekend mortality effects across the 66 study conditions that was explained by the study exposure factors. The weekend mortality effects for each study disorder (based on logistic regression odds ratios) were the continuous outcome measures and the study exposure measures were the explanatory factors. Apart from the categorical medical disease group, the other explanatory variables were measured continuously and were also fitted quadratically in the regression models to allow for non-linear relationships with weekend effects (see Appendix, section II for further details of their measurement). Each explanatory factor was first assessed individually; and then, secondly, in a multivariable analysis using a parsimonious stepwise refinement approach so that only significant terms were retained in the final models. Patient sex was missing in < 0.01% of cases (986 in England and 22 in Wales) and social deprivation in respectively 1.4% (204521) and 2.3% (21184) of cases. Spell duration was complete in Wales but missing in < 0.001% (22) cases in England. There were no missing data for patient age, principal diagnosis, source of admission, date of admission or date of death. With little or no influence on the study findings, missing data were excluded from the analyses involving the respective factors.

Other methods of analysis include mortality and hospital admission rates, Mann-Whitney tests to compare lengths of stay for patients admitted on week days and weekends, and t-tests to compare mean patient ages and numbers of co-morbidities. When comparing mortality according to the week day of admission, mortality rates were standardised using the direct method and the total population of patients admitted as the standard populations. Pearson’s correlations were also used to assess correlations between weekend effect sizes and reductions in admission rates at weekends. Mortality rates were calculated using the numbers of deaths at 30 days as numerators, the numbers of admissions as denominators and were expressed as percentages. Admission rates were calculated using the numbers of admissions as numerators, the resident populations of England and Wales (based on the mid study year) as the denominators and were expressed per 100,000 population.

## Results

For the 66 study disorders overall, there were a total of 14,168,443 hospitalised cases in England and 913,068 in Wales. The mean age of the patients was 66.0 years (SD = 20.4) in England and 66.7 years (SD = 19.7) in Wales. A majority of the patients (52.1% in England and 52.5% in Wales) were female.

### Comparison of patients admitted on week days and weekends

For each of the 66 study disorders, Table 1 shows the numbers of admissions, population admission rates, median lengths of stay, mean ages and numbers of co-morbidities for patients admitted on weekends, compared with week days, in England and in Wales. In both countries, admission rates were lower on weekends for 61 of the 66 study disorders (Table 1). The five exceptions in both countries were all trauma conditions; traumatic brain injury, other head injury, shoulder & upper arm injury, thoracic & abdominal injury and drug poisoning. The conditions with greatest reductions in admission rates at weekends (> 40% reduction compared with week days) in both countries were:

-11 of the 13 study cancers
-four of 13 circulatory diseases (phlebitis & thrombophlebitis, peripheral vascular disease, arterial embolism & thrombosis and hypertension)
-pleural effusion of the 8 respiratory diseases
-alcoholic liver disease and other liver disease of the 12 gastrointestinal disorders
-Alzheimer’s disease of the 3 ageing-related disorders
-none of the 7 trauma conditions.
-anaemia and pressure lower limb ulcers of the other 10 disorders

**Table 1:** Patients admitted on weekends and on week days for major acute disorders across England and Wales, compared according to population admission rates, patient age, inpatient stay and number of patient co-morbidities

	England								Wales							
	Admission rate (per 100,000 population)		Mean patient age (years)		Median inpatient stay (days)		Mean number of patient co-morbidities		Admission rate (per 100,000 population)		Mean patient age (years)		Median inpatient stay (days)		Mean number of patient co-morbidities	
	Week day	Week end	Week day	Week end	Week day	Week end	Week day	Week end	Week day	Week end	Week day	Week end	Week day	Week end	Week day	Week end
Circulatory diseases:																
Acute myocardial infarction	127	116	70.3	70.1	6.0	5.0	1.8	1.8	142	125	70.9	70.3	6.0	5.0	1.8	1.8
Stroke	174	153	75.2	75.7	9.0	10.0	2.0	1.9	190	151	75.5	75.6	10.0	10.0	2.1	2.1
Subarachnoid haemorrhage	8	7	59.3	59.4	4.0	4.0	1.1	1.1	9	7	61.0	60.9	2.0	2.0	1.2	1.1
Heart failure	130	85	78.5	79.3	8.0	7.0	2.3	2.3	162	93	78.4	79.0	8.0	8.0	2.4	2.5
Angina	151	111	68.2	69.0	1.0	2.0	2.1	2.1	177	121	69.7	70.2	2.0	2.0	2.2	2.2
Valvular heart disease	12	8	73.9	75.6	8.0	9.0	1.9	1.9	17	10	74.5	76.6	8.0	9.0	1.9	1.8
Pulmonary embolism	45	28	65.1	66.0	6.0	7.0	1.3	1.3	44	26	64.6	65.3	7.0	8.0	1.3	1.4
Atrial fibrillation	132	89	70.7	70.4	2.0	2.0	1.4	1.4	160	95	71.1	70.5	3.0	3.0	1.4	1.5
Abdominal aortic aneurysm	9	6	77.1	77.6	5.0	4.0	2.1	2.0	10	7	76.7	71.1	5.0	3.0	2.2	2.0
Peripheral vascular disease	11	4	71.4	73.0	7.0	6.0	2.2	2.3	17	6	72.1	71.4	9.0	9.0	2.4	2.3
Arterial embolism & thrombosis	10	5	70.9	72.2	9.0	9.0	2.1	2.2	11	5	70.4	71.7	10.0	9.0	2.1	2.3
Phlebitis & thrombophlebitis	59	20	61.8	59.7	0.0	2.0	1.3	1.3	42	18	63.5	63.0	2.0	3.0	1.5	1.5
Hypertension	27	15	63.2	65.1	2.0	2.0	1.6	1.6	34	16	65.4	67.3	2.0	3.0	1.6	1.7
Respiratory diseases:																
COPD	219	184	71.8	71.9	5.0	4.0	2.2	2.2	296	224	71.7	71.6	6.0	5.0	2.3	2.3
Pulmonary oedema	5	4	74.5	75.3	5.0	5.0	3.2	3.2	6	5	76.1	76.8	6.0	5.0	3.3	3.3
Pneumonia	261	233	72.5	73.1	7.0	6.0	2.5	2.5	281	228	71.8	72.4	7.0	6.0	2.5	2.5
Pneumonitis	21	21	75.7	75.7	11.0	10.0	2.4	2.4	21	19	76.1	75.3	12.0	10.0	2.5	2.4
Lower respiratory infections	145	122	68.8	70.0	4.0	4.0	2.2	2.2	192	145	69.3	69.7	4.0	4.0	2.3	2.4
Pleural effusion	30	14	70.6	71.1	6.0	7.0	2.4	2.5	32	14	71.0	72.1	7.0	8.0	2.6	2.6
Other interstitial respiratory disease	10	6	73.4	74.4	7.0	7.0	2.2	2.2	14	7	74.3	75.2	8.0	8.0	2.2	2.2
Respiratory failure	11	9	69.9	70.5	8.0	8.0	2.7	2.7	9	6	69.1	69.0	9.0	10.0	2.7	2.6
Gastrointestinal disorders:																
Upper gastrointestinal bleeding	66	53	61.6	60.8	3.0	3.0	1.9	1.8	69	50	62.1	61.1	3.0	3.0	1.9	1.9
Perforated peptic ulcer & peritonitis	12	10	62.2	61.7	8.0	8.0	1.8	1.8	11	9	64.3	64.2	9.0	9.0	2.0	1.8
Diverticular disease	46	33	67.9	68.8	5.0	4.0	1.8	1.9	62	41	68.1	68.8	5.0	4.0	1.9	2.0
Intestinal obstruction	40	32	67.3	67.4	6.0	6.0	1.9	1.9	40	32	67.7	67.9	6.0	5.0	2.0	1.9
Herniae	39	25	63.1	64.4	2.0	3.0	1.6	1.6	47	28	63.3	65.5	2.0	4.0	1.7	1.8
Alcoholic liver disease	27	15	51.6	51.4	9.0	9.0	1.7	1.8	37	18	52.3	51.9	9.0	9.0	1.6	1.6
Other liver disease	15	9	60.0	59.5	8.0	8.0	2.0	2.1	16	9	61.6	61.2	8.0	9.0	2.0	2.0
Gallstone disease	103	82	58.1	57.8	4.0	4.0	1.4	1.3	145	111	58.2	57.6	4.0	4.0	1.4	1.3
Acute pancreatitis	35	32	54.9	55.2	5.0	5.0	1.5	1.5	37	31	56.6	56.8	6.0	5.0	1.5	1.5
Intestinal infections	46	40	60.6	60.2	4.0	3.0	1.8	1.8	47	37	62.0	61.0	4.0	4.0	2.0	2.0
Noninfective gastroenteritis	90	77	59.5	59.4	2.0	2.0	1.8	1.7	111	81	60.7	60.4	6.0	5.0	1.9	1.8
Constipation	60	48	64.4	65.7	2.0	2.0	1.8	1.9	79	57	64.0	65.4	2.0	2.0	1.9	2.0
Trauma:																
Hip fracture	129	120	80.9	80.5	15.0	15.0	2.0	2.0	145	130	80.7	79.8	15.0	13.0	2.2	2.1
Traumatic brain injury	20	24	58.6	51.4	4.0	3.0	1.3	1.0	17	19	59.8	53.1	5.0	4.0	1.5	1.1
Other head injury	135	203	55.3	47.2	1.0	1.0	1.1	0.8	98	161	52.1	43.8	1.0	1.0	1.1	0.7
Shoulder & upper arm injury	56	58	64.1	59.9	2.0	2.0	1.0	1.0	54	56	63.5	57.9	3.0	3.0	1.4	1.2
Thoracic & abdominal injury	50	58	54.9	50.1	2.0	2.0	1.1	1.0	49	57	55.3	49.7	3.0	3.0	1.2	1.0
Fracture of lumbar spine & pelvis	28	26	71.5	67.9	8.0	9.0	1.6	1.5	33	30	72.0	67.2	10.0	10.0	1.8	1.6
Drug poisoning	175	193	38.1	36.8	1.0	1.0	0.7	0.6	180	211	37.9	36.4	1.0	1.0	0.7	0.6
Cancers:																
Oesophageal	14	7	71.2	71.2	7.0	7.0	1.5	1.5	18	8	71.3	71.6	8.0	7.0	1.5	1.5
Gastric	10	6	72.8	72.6	8.0	8.0	1.5	1.5	15	7	73.5	72.8	9.0	10.0	1.5	1.5
Colorectal	34	20	71.5	71.7	10.0	11.0	1.6	1.5	46	24	72.6	72.1	12.0	11.0	1.6	1.5
Pancreatic	13	7	71.3	71.0	10.0	10.0	1.6	1.5	17	7	71.7	71.9	10.0	10.0	1.6	1.5
Liver	6	3	70.0	69.6	10.0	10.0	2.0	2.1	7	3	71.6	71.4	11.0	9.0	2.0	2.0
Breast	16	9	62.2	62.2	5.0	5.0	0.9	0.9	14	7	67.9	67.3	8.0	6.0	1.2	1.2
Lung	50	31	70.8	71.2	8.0	8.0	1.6	1.7	68	34	71.1	71.4	9.0	9.0	1.7	1.8
Prostate	16	9	76.2	76.7	7.0	7.0	1.6	1.7	23	12	76.9	77.0	8.0	8.0	1.7	1.7
Ovarian	9	5	66.0	65.9	7.0	7.0	1.1	1.1	10	5	67.2	65.7	9.0	8.0	1.2	1.1
Lymphomas	47	22	64.6	64.4	6.0	7.0	1.4	1.5	48	21	65.9	65.7	6.0	6.0	1.4	1.5
Bladder	10	6	75.9	76.5	9.0	9.0	2.0	2.0	13	7	76.3	76.6	10.0	9.0	2.0	2.3
Kidney	5	3	68.7	69.1	8.0	9.0	1.6	1.6	7	3	70.5	70.0	10.0	10.0	1.6	1.7
Brain	9	6	60.0	59.7	9.0	8.0	1.0	0.9	9	5	60.7	60.1	9.0	9.0	1.1	1.1
Ageing-related disorders:																
Alzheimer’s disease	10	6	80.8	81.6	28.0	17.0	2.2	2.3	17	6	81.2	82.0	31.0	22.0	2.2	2.4
Dementia	28	18	82.3	83.3	24.0	17.0	1.7	1.8	46	19	82.0	83.0	32.0	22.0	1.7	1.8
Age-related physical debility	57	50	83.8	84.1	6.0	5.0	2.4	2.4	54	40	83.7	83.9	10.0	9.0	2.7	2.6
Other acute disorders:																
Urinary tract infections	251	223	67.8	68.8	4.0	4.0	2.1	2.1	261	206	67.3	67.5	4.0	5.0	2.1	2.1
Acute renal failure	44	27	74.8	75.7	8.0	9.0	2.6	2.6	44	24	75.0	75.1	8.0	9.0	2.7	2.7
Diabetes	68	45	53.4	51.1	3.0	3.0	1.7	1.6	83	49	55.6	52.6	5.0	4.0	1.9	1.8
Septicaemia	41	36	71.8	72.2	8.0	7.0	2.6	2.6	52	41	71.9	72.2	8.0	7.0	2.7	2.7
Skin infections	196	130	53.5	52.2	2.0	2.0	1.4	1.3	197	121	54.2	53.2	3.0	3.0	1.5	1.4
Anaemias	87	35	66.6	62.0	2.0	3.0	2.2	2.1	95	28	71.6	71.7	4.0	5.0	2.5	2.5
Pressure lower limb ulcers	24	10	71.3	71.7	11.0	11.0	2.6	2.6	30	9	73.1	73.8	13.0	13.0	2.8	2.8
Disorientation - unspecified	32	26	76.8	76.6	5.0	4.0	2.3	2.2	32	25	77.7	76.9	7.0	5.0	2.6	2.5
Malaise & fatigue	19	13	65.3	66.5	1.0	1.0	1.9	2.0	22	12	70.9	72.0	3.0	3.0	2.2	2.2
Syncope & collapse	142	137	67.7	68.0	1.0	1.0	1.9	1.9	150	140	68.1	68.3	1.0	1.0	2.1	2.0

For all 66 conditions combined, patients admitted on weekends, compared with week days, were slightly younger (65.0 vs 66.3 years in England and 65.2 vs 67.2 in Wales). They had slightly shorter lengths of inpatient stay when admitted on weekends (median = 4.0 vs 4.0 days in England and 4.0 vs 5.0 in Wales) and slightly lower numbers of co-morbidities recorded (mean 2.1 vs 2.2 in England and 2.1 vs 2.3 in Wales). With the very large study sizes, all of these slight differences were significant (*p* < 0.001). Patients with head and upper body injuries were much younger when admitted at weekends compared with week days (by mean of 4 to 9 years; *p* < 0.001), while patients admitted at weekends for Alzheimer’s disease and dementia had much shorter inpatient stays (by median of 7 to 11 days; *p* < 0.001; Table 1).

For almost all disorders, as expected, patients admitted at weekends were more often via hospital emergency departments rather than through primary care or consultant clinics, but had similar levels of social deprivation (Additional file 1: Table S1). The highest levels of social deprivation (> 50% of patients admitted in the most deprived quintiles IV and V in England) were seen in people with alcoholic liver disease, drug poisoning, COPD, respiratory failure, acute pancreatitis, head injuries and diabetes. The lowest levels of deprivation (< 38% in quintiles IV and V) were for atrial fibrillation, lymphomas and colorectal, pancreatic, prostate, ovarian and brain cancers.

### Mortality and admission rates according to the day of the week

**Fig. 1:**
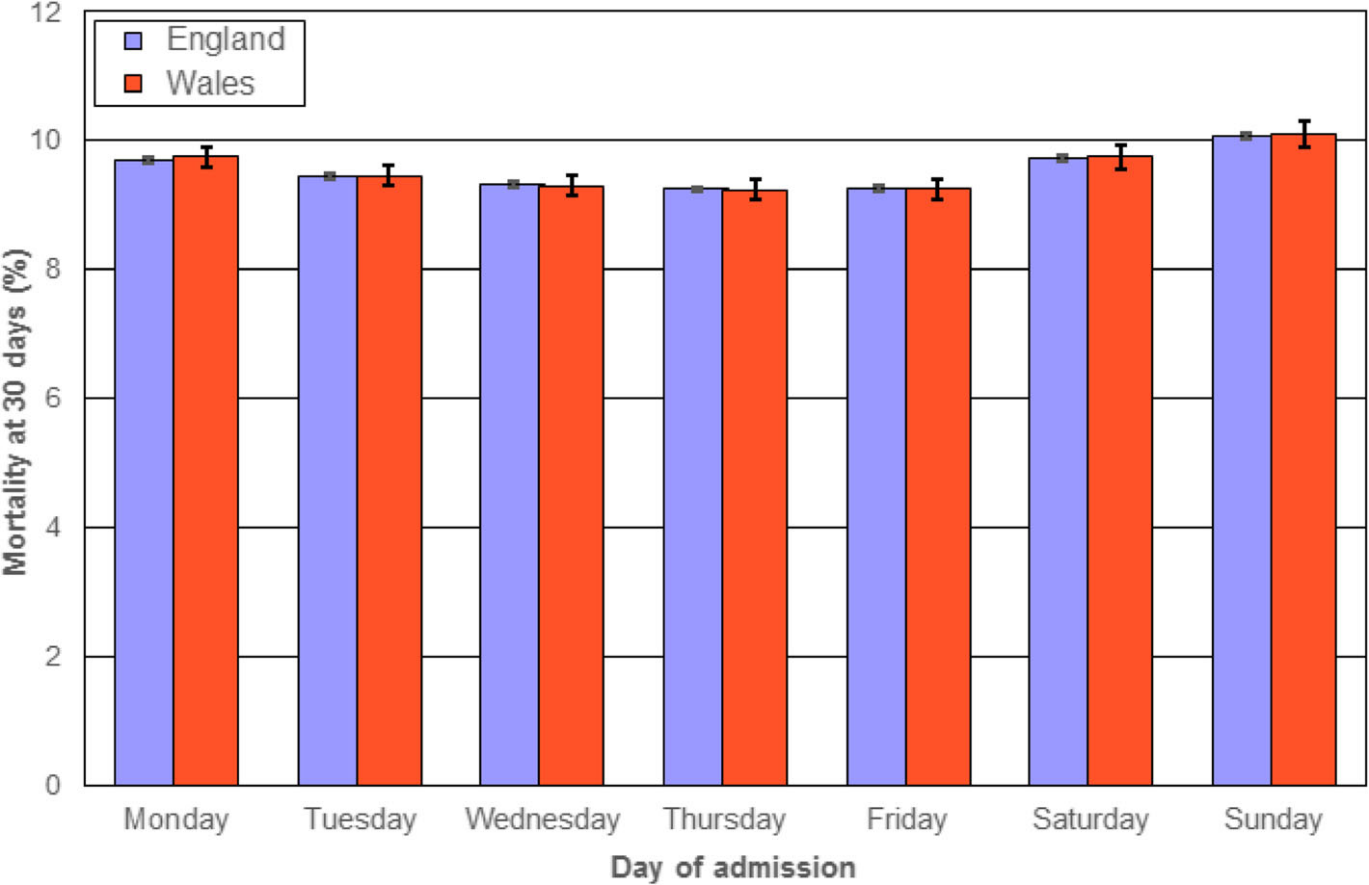
Standardised mortality rates (at 30 days) for all 66 study conditions combined according to the week day of admission in England and Wales. Notes Mortality rates are standardised for age group and sex. Vertical bars represent 95% confidence intervals. Overall admission rates for the 66 conditions (per 1000 population) for each day of the week in England and Wales respectively were: Monday 42.9; 48.5, Tuesday 41.0; 45.9, Wednesday 40.0; 44.8, Thursday 40.3; 45.1, Friday 41.6; 47.0, Saturday 33.1; 34.2, Sunday 32.7; 33.2

Figure 1 shows overall mortality rates for admissions on each day of the week, with corresponding admission rates denoted in the footnote to Fig. 1. In both England and Wales, mortality rates were highest for admissions on Sundays, followed by Saturdays and Mondays. Admission rates were lowest on Saturdays and Sundays and highest on Mondays, but mortality rates were higher on Mondays than on other week days.

### Overall weekend mortality effects

There were significant weekend effects (of any size) for 46 of the 66 study conditions in England and for 21 in both England and Wales (Table 2). There were also large consistent weekend effects (> 20%) for 22 of the 66 conditions in England. These were for 8 of the 13 study cancers, circulatory diseases (5 of 12), respiratory (2 of 8), gastrointestinal (3 of 12), ageing-related (2 of 3) and other disorders (2 of 10). For 14 of these 22 conditions, there were also large significant weekend effects (> 20%) in the second population, Wales.

**Table 2:** Numbers of admissions, mortality rates, weekend mortality effects and reductions in admission rates at weekends for major acute disorders across England and Wales

	England				Wales			
	Number of admissions	Mortality rate (30 days)	% increased mortality for weekend admissions (based on odds ratios, 95% CI)	% reduced admission rate at weekends	Number of admissions	Mortality rate (30 days)	% increased mortality for weekend admissions (based on odds ratios, 95% CI)	% reduced admission rate at weekends
Circulatory diseases:								
Acute myocardial infarction	462 983	12.3%	5.9 (3.7, 8.2)	9	30 045	13.7%	4.0 (-4.0, 12.6)	12
Stroke	627 553	19.8%	11.5 (9.9, 13.2)	12	38 954	20.5%	19.3 (12.5, 26.5)	20
Subarachnoid haemorrhage	28 699	32.0%	13.5 (6.8, 20.6)	9	1765	35.5%	25.2 (-2.1, 60.1)	16
Heart failure	434 672	17.1%	13.4 (11.2, 15.6)	34	30 908	17.2%	9.2 (1.1, 17.8)	**43**
Angina	519 591	1.5%	**22.1** (15.9, 28.5)	26	35 065	2.0%	**23.8** (4.0, 47.3)	32
Valvular heart disease	41 644	7.8%	13.0 (3.3, 23.7)	37	3288	7.3%	19.0 (-14.0, 64.7)	**43**
Pulmonary embolism	149 107	8.3%	19.7 (14.4, 25.2)	37	8394	7.9%	**24.5** (2.1, 51.8)	**40**
Atrial fibrillation	445 430	2.3%	15.9 (10.5, 21.6)	32	30 726	1.9%	3.0 (-16.5, 27.0)	**41**
Abdominal aortic aneurysm	29 988	39.5%	**51.0** (42.4, 60.1)	27	1984	40.9%	**94.5** (54.8, 144)	27
Peripheral vascular disease	33 025	13.4%	**49.4** (36.5, 63.7)	**58**	3000	10.9%	**89.7** (36.1, 164)	**67**
Arterial embolism & thrombosis	30 161	9.8%	**34.4** (21.5, 41.8)	**51**	2042	12.0%	**113** (51.2, 199)	**52**
Phlebitis & thrombophlebitis	175 390	2.1%	**49.3** (36.4, 63.5)	**66**	7612	2.6%	8.7 (-26.7, 61.1)	**58**
Hypertension	87 451	5.1%	17.5 (8.7, 26.9)	**47**	6305	4.9%	1.5 (-26.2, 39.5)	**53**
Respiratory diseases:								
COPD	783 360	7.6%	3.5 (1.5, 5.6)	16	60 472	7.1%	6.7 (-1.0, 15.0)	24
Pulmonary oedema	18 665	15.7%	1.2 (-8.1, 11.5)	14	1271	19.0%	10.2 (-22.0, 55.6)	15
Pneumonia	950 207	25.3%	3.7 (2.5, 4.9)	11	58 392	24.5%	9.2 (4.3, 14.5)	19
Pneumonitis	79 303	45.7%	0.8 (-2.5, 4.2)	4	4388	48.2%	**22.9** (11.7, 35.2)	11
Lower respiratory infections	520 206	7.7%	5.7 (3.1, 8.3)	16	39 158	6.9%	0.1 (-9.0, 10.1)	24
Pleural effusion	94 263	12.3%	**41.5** (34.3, 49.2)	**54**	5778	13.1%	**44.6** (17.6, 77.9)	**57**
Other interstitial respiratory disease	33 787	24.6%	**42.4** (33.8, 51.5)	38	2584	24.3%	21.2 (-4.2, 53.4)	**46**
Respiratory failure	37 538	37.2%	7.8 (2.2, 13.7)	17	1746	36.9%	3.6 (-19.8, 33.8)	32
Gastrointestinal disorders:								
Upper gastrointestinal bleeding	232 762	7.7%	9.9 (6.0, 14.1)	19	13 861	7.5%	-3.5 (-17.7, 13.2)	27
Perforated peptic ulcer & peritonitis	42 303	22.4%	8.0 (1.7, 14.6)	19	2365	25.4%	-0.3 (-22.2, 27.7)	20
Diverticular disease	158 390	3.9%	11.7 (5.1, 18.8)	28	12 171	3.2%	13.1 (-11.8, 45.0)	33
Intestinal obstruction	139 882	11.0%	7.8 (3.5, 12.3)	19	8264	11.7%	2.5 (-13.3, 21.1)	22
Herniae	128 755	3.3%	**27.7** (18.6, 37.6)	36	9213	3.5%	13.2 (-14.4, 49.9)	**40**
Alcoholic liver disease	88 589	17.8%	**27.4** (21.8, 33.3)	**45**	6821	16.9%	**26.2** (6.1, 50.0)	**53**
Other liver disease	48 405	15.8%	**26.4** (18.7, 34.6)	**42**	3066	16.7%	**37.1** (6.9, 75.8)	**47**
Gallstone disease	361 616	1.2%	-0.2 (-7.2, 7.3)	20	29 498	1.2%	24.2 (-3.5, 59.7)	24
Acute pancreatitis	128 885	4.1%	3.3 (-20.1, 33.7)	10	7686	4.9%	3.9 (-19.7, 34.3)	16
Intestinal infections	166 662	4.4%	-5.4 (-10.6, 0.1)	13	9561	4.6%	-11.8 (-30.9, 12.5)	21
Noninfective gastroenteritis	322 727	3.2%	3.9 (-0.8, 8.8)	15	21 811	2.9%	15.0 (-4.8, 38.9)	27
Constipation	211 598	3.1%	0.6 (-5.1, 6.6)	20	15 835	2.9%	6.3 (-15.1, 33.1)	28
Trauma:								
Hip fracture	472 153	8.0%	1.9 (-0.6, 4.4)	7	30 786	7.3%	8.6 (-1.7, 20.0)	10
Traumatic brain injury	79 496	14.4%	6.5 (1.7, 11.4)	-23	3818	16.2%	12.8 (-7.6, 37.8)	-14
Other head injury	579 485	1.3%	2.3 (-2.8, 7.7)	-51	25 448	1.1%	-4.8 (-27.4, 24.8)	-64
Shoulder & upper arm injury	211 342	2.0%	5.5 (-1.6, 13.0)	-5	11 967	1.7%	-16.9 (-41.0, 17.2)	-2
Thoracic & abdominal injury	196 541	2.1%	1.5 (-5.4, 11.9)	-16	11 283	1.8%	-3.9 (-30.7, 33.1)	-16
Fracture of lumbar spine & pelvis	102 544	3.4%	9.4 (1.2, 18.3)	5	7033	3.3%	-14.1 (-38.4, 19.6)	10
Drug poisoning	672 803	0.7%	-5.5 (-11.9, 1.4)	-10	41 374	0.6%	-6.5 (-29.8, 24.7)	-17
Cancers:								
Oesophageal	44 574	29.1%	**29.4** (22.7, 36.4)	**47**	3291	28.9%	**34.2** (8.8, 65.6)	**57**
Gastric	33 861	30.3%	18.5 (14.3, 22.8)	**45**	2798	31.8%	**39.7** (11.7, 74.8)	**57**
Colorectal	111 162	23.0%	**26.2** (18.9, 34.1)	**42**	8532	23.6%	**26.0** (10.4, 43.9)	**48**
Pancreatic	41 646	37.0%	**28.8** (22.0, 35.8)	**48**	3034	36.3%	20.0 (-3.0, 48.6)	**56**
Liver	17 915	36.5%	**28.8** (15.2, 35.5)	**47**	1197	38.3%	36.3 (-2.0, 89.4)	**55**
Breast	51 353	23.8%	19.0 (12.8, 25.5)	**44**	2634	29.8%	4.3 (-16.8, 30.8)	**47**
Lung	165 263	39.7%	**28.1** (24.9, 31.3)	39	12 691	41.0%	**22.9** (11.7, 35.2)	**49**
Prostate	53 016	24.5%	**20.2** (14.3, 26.5)	**46**	4262	24.6%	3.8 (-13.9, 25.2)	**48**
Ovarian	29 024	22.4%	**29.9** (20.6, 40.0)	**50**	1838	25.6%	20.0 (-10.5, 60.9)	**50**
Lymphomas	147 400	16.4%	**44.4** (39.1, 49.9)	**53**	8716	17.3%	**30.9** (11.9, 53.0)	**57**
Bladder	33 738	21.6%	5.0 (-1.7, 12.3)	**40**	2489	21.4%	-5.6 (-27.5, 22.9)	**46**
Kidney	17 723	25.8%	8.0 (-1.1, 17.9)	**43**	1348	25.9%	14.5 (-19.8, 61.4)	**53**
Brain	29 276	17.0%	17.2 (8.9, 26.0)	29	1773	18.2%	-22.6 (-44.9, 8.7)	**44**
Ageing-related disorders:								
Alzheimer's disease	32 124	9.3%	**36.2** (24.3, 49.3)	**43**	3027	7.8%	**66.0** (16.5, 136)	**63**
Dementia	92 558	10.0%	**24.6** (18.1, 31.3)	37	8450	6.8%	**48.0** (19.8, 82.8)	**59**
Age-related physical debility	204 767	5.2%	**-5.8** (-10.0, -1.3)	12	10 899	4.5%	6.4 (-14.3, 32.1)	26
Other acute disorders:								
Urinary tract infections	907 855	4.5%	0.4 (-1.9, 2.7)	11	53 585	3.8%	3.1 (-7.2, 14.6)	21
Acute renal failure	147 012	23.3%	**31.4** (27.4, 35.5)	39	8238	24.1%	**20.3** (5.1, 37.6)	**45**
Diabetes	229 430	2.5%	**25.9** (18.1, 34.2)	34	16 036	2.4%	14.0 (-12.1, 47.9)	**41**
Septicaemia	147 156	34.9%	2.0 (-0.7, 4.7)	13	10 611	34.6%	6.1 (-4.0, 17.3)	21
Skin infections	657 895	1.3%	10.8 (5.0, 17.0)	34	38 083	1.2%	18.4 (-5.8, 49.0)	39
Anaemias	266 850	3.8%	18.9 (12.2, 16.1)	**60**	16 034	4.2%	**31.0** (3.4, 66.0)	**71**
Pressure lower limb ulcers	75 760	7.4%	17.6 (9.1, 26.8)	**57**	5146	6.9%	**44.4** (5.4, 91.7)	**70**
Disorientation - unspecified	113 080	4.4%	-3.6 (-9.9, 3.2)	17	6321	4.6%	19.2 (-10.2, 58.3)	21
Malaise & fatigue	65 050	5.4%	-5.3 (-13.0, 3.2)	30	4113	7.6%	7.1 (-21.0, 45.3)	**46**
Syncope & collapse	524 994	2.2%	-1.9 (-5.9, 2.2)	4	32 184	1.9%	1.9 (-15.2, 22.4)	7

Bold values for % increased mortality for weekend admissions denote large significant weekend mortality effects (>20%)

Bold values for reduced admissions at weekends denote large reductions (>40%)

Weekend mortality effects are based on odds ratios, obtained through multiple logistic regression modelling

### Circulatory disease and weekend mortality effects

**Fig. 2:**
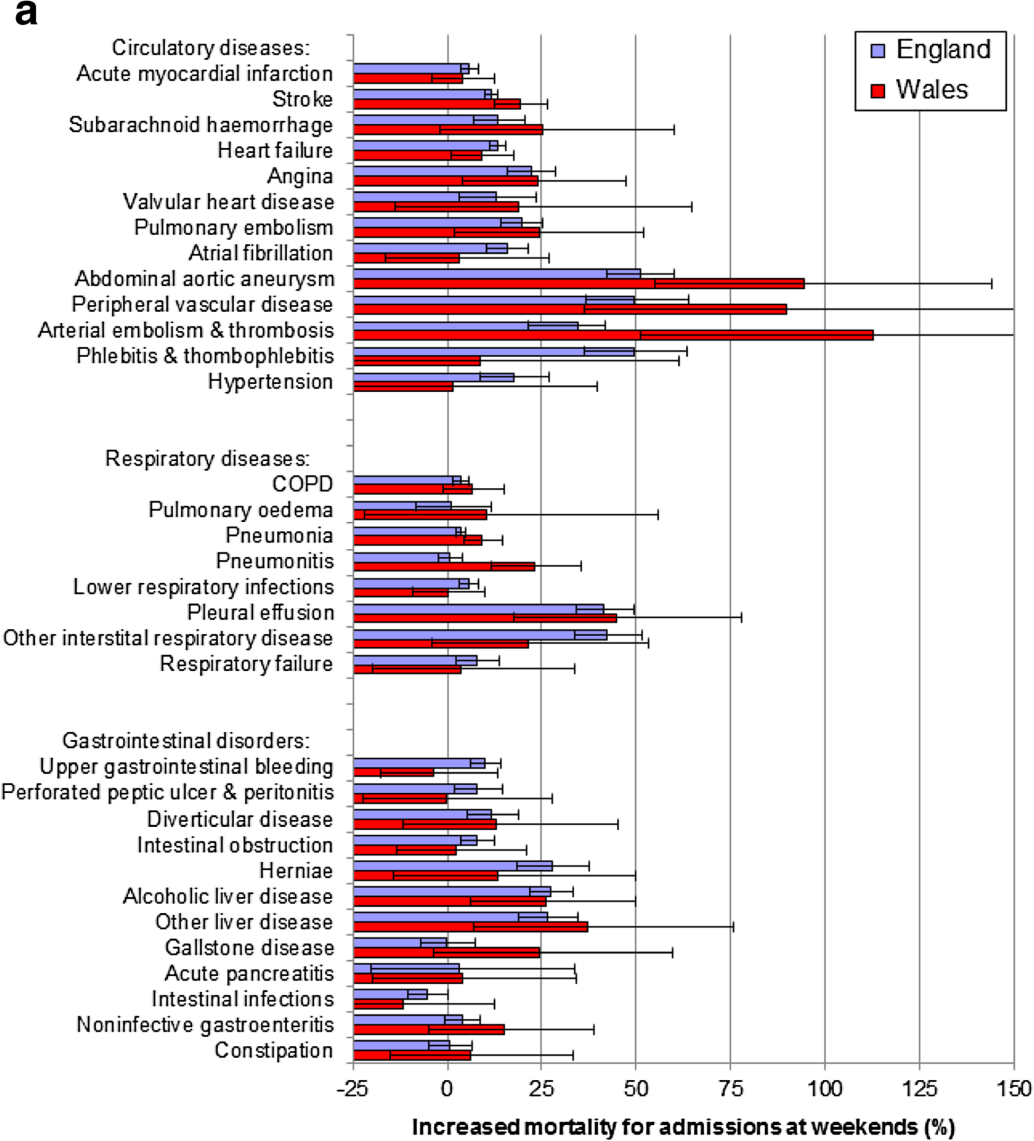

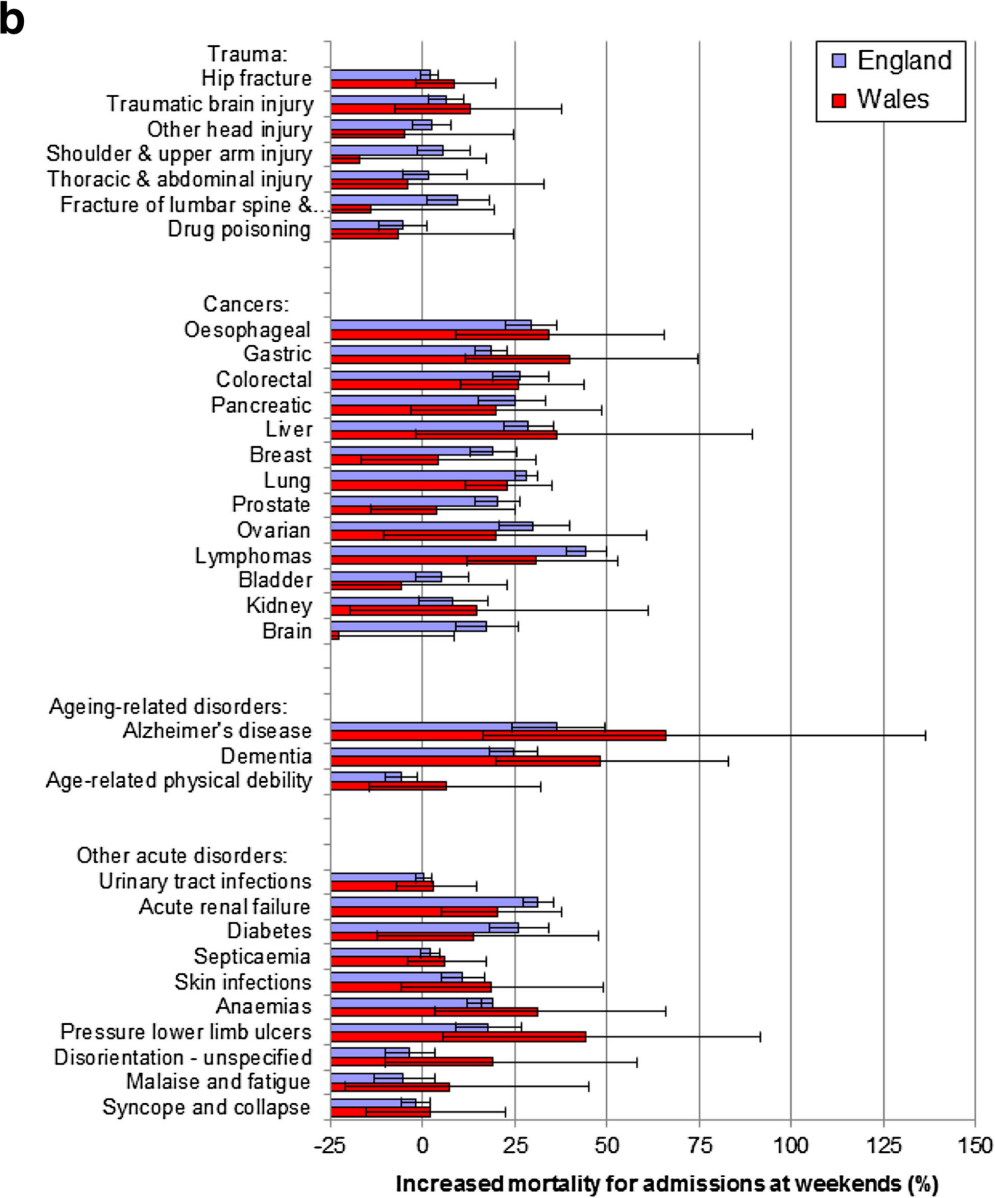
**a** Weekend mortality effects for acute circulatory, respiratory and gastrointestinal disorders across England and Wales. Notes. The thick bold vertical line at 0 denotes no weekend mortality effect. Horizontal bars represent 95% confidence intervals. The increased mortality for admissions at weekends (%) are based on odds ratios, obtained through multiple logistic regression modelling. **b** Weekend mortality effects for trauma, cancers, ageing-related and other acute disorders across England and Wales. Notes. The thick bold vertical line at 0 denotes no weekend mortality effect. Horizontal bars represent 95% confidence intervals. The increased mortality for admissions at weekends (%) are based on odds ratios, obtained through multiple logistic regression modelling

Of the 13 circulatory disorders, there were large weekend effects (> 20%) in England for 5 (angina, 22.1%; abdominal aortic aneurysm, 51.0%; peripheral vascular disease, 49.4%; arterial embolism & thrombosis, 34.4%; and phlebitis and thrombophlebitis, 49.3%; Table 2; Fig. 2a). There were also large weekend effects in both countries for the first 4 of these conditions. Additionally, weekend effect sizes for pulmonary embolism were 19.7% in England and 24.5% in Wales. For most other circulatory diseases, there were smaller but significant weekend effects of 10 to 20% in England.

### Respiratory diseases and weekend mortality effects

There were large weekend effects in England for 2 of the 8 respiratory disorders, pleural effusion and other interstitial respiratory disease (Table 2; Fig. 2a) but a large weekend effect in both countries only for pleural effusion.

### Gastrointestinal disorders and weekend mortality effects

There were large weekend effects in England for 3 of the 12 gastrointestinal disorders (alcoholic liver disease, other liver disease and herniae) and large weekend effects in both populations for the first two.

### Trauma and weekend mortality effects

There were no large weekend effects for any of the 7 trauma conditions in England, or in Wales, and only small significant weekend effects for two trauma conditions (traumatic brain injury and fracture of lumbar spine & pelvis, 9.4%) in England (Table 2; Fig. 2b).

### Cancers and weekend mortality effects

Eight cancers had large weekend effects in England (oesophageal, gastric, colorectal, pancreatic, liver, lung, prostate, ovarian and lymphomas) and 4 in both populations (oesophageal, colorectal, lung and lymphomas). There were also significant weekend effects of > 15% for all but 2 of the 13 cancers in England (Table 2; Fig. 2b).

### Ageing-related disorders and weekend mortality effects

Of the 3 ageing-related disorders, there were large consistent mortality effects in England and also in Wales for 2 (Alzheimer’s disease and dementia) but an inverse weekend effect in England for age-related physical debility.

### Other disorders and weekend mortality effects

Of the remaining 10 study disorders, two (diabetes and acute renal failure) had large weekend effects in England, but only acute renal failure in both countries. Three others (skin infections, anaemias and pressure lower limb ulcers) had smaller significant weekend effects in England.

### Explanation of study exposure measures on weekend mortality effects

For the study exposure measures, assessed individually, variation in weekend effects across the 66 disorders was explained most strongly in England by reductions in admission rates at weekends (58.1%; *p* < 0.001), followed by the medical disease category (25.3%; *p* = 0.007) and changes in the source of admissions at weekends (hospital emergency departments vs primary care and consultant clinics; 27.2%; *p* < 0.001; Table 3). When the factors were assessed simultaneously in multivariable analysis, reductions in admissions at weekends and the medical disease category had independent influence on variation in the weekend mortality effects (combined explanation = 65.4%, reductions in admissions alone = 56.5%) but changes in the source of admission was no longer significant. In Wales, reductions in admission rates and medical disease category explained respectively 27.8 and 22.6% of the variation in weekend mortality effects individually and 40.2% when combined. When weighted and combined across England and Wales, reductions in admission rates and medical disease category explained 64.0% of the variation in weekend mortality effects.

**Table 3:** Variation in weekend mortality effects across the 66 study disorders, explained by study exposure measures

	Variation in weekend mortality effects explained	
Exposure measure (assessed individually)	England	Wales
Medical disease category	25.3% (*p* = 0.007)	22.6% (*p* = 0.016)
Reductions in admission rates at weekends	58.1% (*p* < 0.001)	27.8% (*p* < 0.001)
Deaths on the day of admission	20.3% (*p* < 0.001)	15.8% (*p* = 0.004)
Mean patient age	4.9% (*p* = 0.199)	9.6% (*p* = 0.040)
Patient social deprivation	4.9% (*p* = 0.201)	2.3% (*p* = 0.476)
Changes in the source of admission at weekends (through hospital emergency departments vs primary	27.1% (*p* < 0.001)	9.3% (*p* = 0.135)

Notes

See the Methods section and Appendix, section II for further details of the exposure measures

**Fig. 3:**
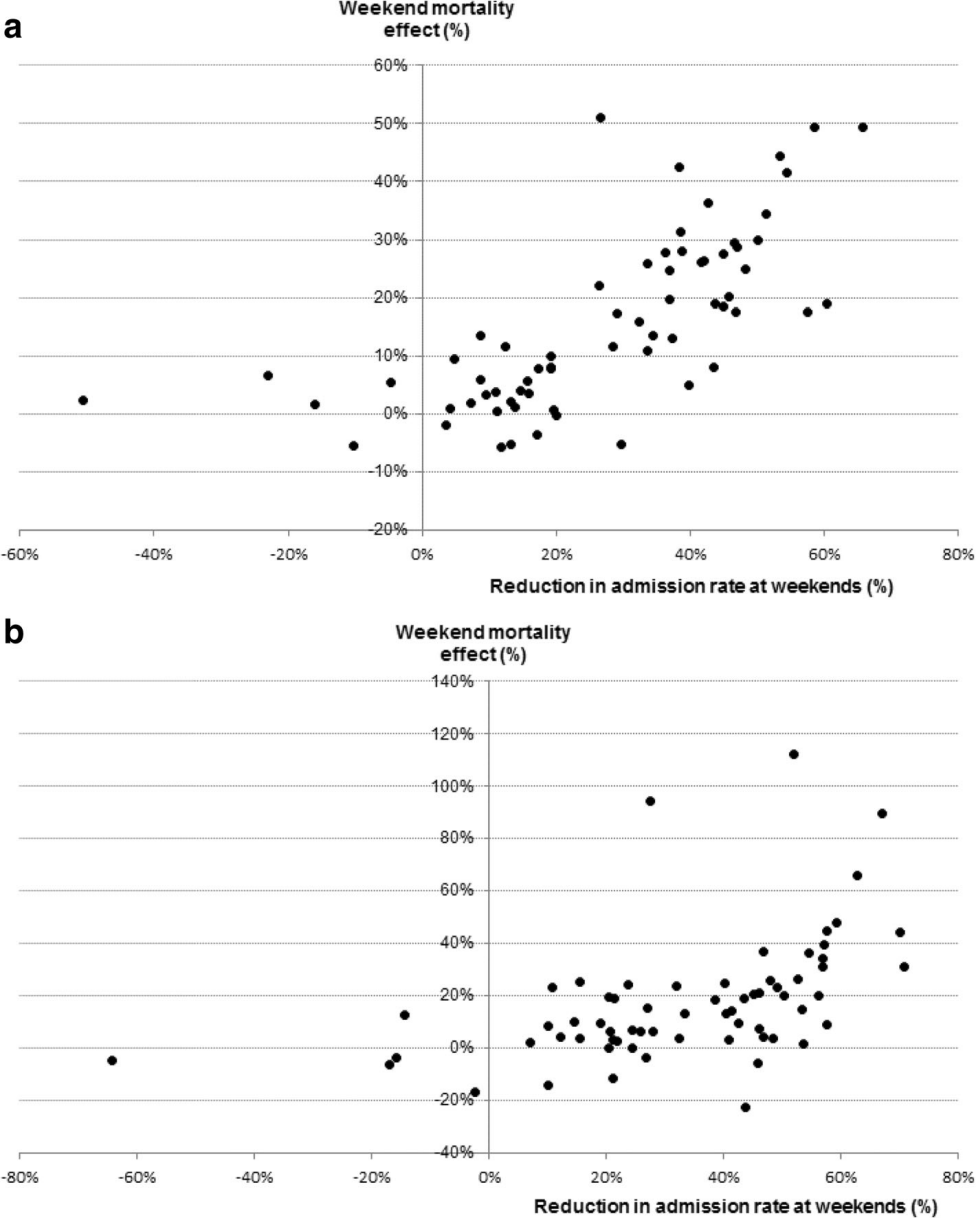
Weekend mortality effects in relation to percentage reductions in admission rates for each of the 66 study disorders in: **a**). England, **b**).Wales

Figure 3 shows strong positive correlations between the weekend effects and the percentage reductions in the admission rate at weekends in England (Fig. 3a) and Wales (Fig. 3b). Pearson’s correlations are 0.70 in England and 0.47 in Wales (both *p* < 0.001). The wider dispersion in weekend effect sizes in Wales compared with England reflects the much greater study power in England (14.2 vs 0.9 million admissions).

## Discussion

The study was designed to establish and compare weekend mortality effects for all major acute disorders with appreciable 30 day mortality. There were large, statistically significant weekend mortality effects (> 20%) for 22 of the 66 conditions in England and for 14 in both England and Wales. These 14 were for cancers, some (lower immediate risk, mostly less common) circulatory disorders, liver disease and several other conditions that are mostly ageing-related or cancer-related. Reductions in weekend admissions and the medical disease category explained 64% of the variation in weekend effect sizes across the study disorders.

### Cancers

There were significant weekend mortality effects of > 15% in England for all but 2 of the 13 study cancers and large significant weekend effects (> 20%) in both countries for 4 of the 13. These weekend effects are consistent with previous evidence on cancers and weekend admissions. [20, 31, 33, 34, 40–43] Moreover, admission rates for cancers were greatly reduced in both countries at weekends (consistently between 29 and 57% for each cancer and > 40% in all but one case). Reduced availability of hospice care in the community at weekends, [44] could lead to more terminal cases hospitalised, while reduced access to specialist oncology therapy at weekends could lead to more non-terminal cases admitted during week days for treatment. Explanation for the contrasting lack of weekend effects for 2 of the less common cancers, bladder and kidney, may be due to smaller numbers or the clinical presentation of these types.

### Respiratory diseases

Of 8 respiratory disorders, there was a large consistent weekend effect in England and also in Wales only for pleural effusion, with greatly reduced admission rates at weekends (> 40%). Most pleural effusions present with gradual breathlessness so that people can wait 2–3 days to see their specialist. Those attending Emergency Departments at weekends are likely to have more severe and more rapid symptoms, consistent with more advanced, rapidly progressive disease or more co-morbidities. Many pleural effusions are cancer related and most recurrences are gradual and would be referred to cancer services during week days. There were no other large weekend effects for the respiratory conditions, other than for pneumonitis and other interstitial respiratory disease in one country.

### Circulatory diseases

There were large consistent weekend mortality effects (> 20%) in England for 5 of 13 circulatory disorders (angina, abdominal aortic aneurysm, peripheral vascular disease, arterial embolism & thrombosis and phlebitis & thrombophlebitis) and in both countries for the first 4 of these. Abdominal aortic aneurysm has been linked consistently with very large weekend effects. [20, 24, 31, 33–45] It carries exceptionally high very early mortality that requires highly specialist intervention and had among the largest of all weekend effects in this study (51% in England and 95% in Wales).

Stroke, subarachnoid haemorrhage and acute myocardial infarction also carry high mortality during acute phases but mortality rates and weekend mortality effects have fallen over time following extensive auditing and widespread improvements in care over the last 15 years, [46, 47] including increased provision of dedicated stroke units that overcome weekend mortality effects in many hospitals. [7, 8] A lack of a weekend effect for acute myocardial infarction is also reported consistently, [9–12, 20, 24] with effective acute treatment for this condition now standardised with 24/7 dedicated percutaneous angioplasty services available throughout most of the UK.

The other circulatory disorders with large weekend mortality effects, including angina, peripheral vascular disease, arterial embolism & thrombosis and (thrombo) phlebitis, carry lower mortality during acute phases but have greatly reduced admissions at weekends (mostly > 50% lower). Unlike acute MI and stroke, these disorders have not been subject to national audits or service reorganisations that have improved both out of hours service provision and patients outcomes, as well as appearing to reduce weekend effects.

### Gastrointestinal disorders

There were no large, consistent weekend mortality effects for gastrointestinal disorders, except for liver disease and herniae in England, and for liver disease in both countries. Both alcoholic and other liver disease had higher mortality (> 20%) but much lower admission rates (> 40%) at weekends. As well as case selection for essentially chronic diseases that are often terminal when presenting acutely, there is a recognised deficit in specialist hepatology provision in the UK. [48] This is likely to be more apparent for the management of complex cases which may sometimes receive lower priorities in acute care at weekends.

### Trauma

There was no weekend mortality effect for any major trauma condition and an increase in admissions at the weekend for most conditions, especially head injuries, which largely affect younger patients and are often alcohol-related, reflecting the known epidemiology of trauma. [16, 17] Following a series of large scale studies in the US, Australia, Canada and the UK, [49–52] trauma services are now configured to operate on a 24/7 basis to reflect the weekly incidence of serious injuries. Our findings are in keeping with the UK Trauma Audit Research Network study, [17] and other previous studies, [31, 34] which did not find trauma weekend mortality effects.

### Ageing-related disorders

There were large weekend mortality effects and greatly reduced admission rates at weekends for cognitive ageing-related disorders (Alzheimer’s disease and dementia) in England and also in Wales. At weekends these admissions were almost entirely through emergency departments, indicating that pre-hospital selection effects, [53] and reduced availability of social care provision at weekends could lead to higher admission thresholds.

### Strengths and limitations

To provide confirmatory evidence, the study was designed and conducted on similar but independently collected information sources from two health services. It is one of the largest studies to report on the weekend effect, covering more than 14 million acute admissions and 1.3 million deaths in England and almost one million admissions and 90,000 deaths in Wales. It excludes elective admissions which are often for diagnostic investigations rather than for (treatment of) active or present disease, [54] and is based on a validated record linkage methodology, [36, 37] that has been used extensively in previous published studies. The inpatient data sources are confined to public hospitals, but these account for almost all of the acute admissions in the two populations. The 66 study disorders also account for almost half (47%) of all acute admissions in England and Wales during the study period, so that generalisability of findings to all acute admissions is strong. Nothing has been reported previously on weekend mortality for many of the study orders - including Alzheimer’s disease, pleural effusion, pneumonitis, anaemias and pressure lower limb ulcers - so that many of the findings are novel. Although the population of Wales is 17 times smaller than the population of England which affects significance of study findings, we have been able to confirm large weekend effects for many of the conditions identified in England.

The study has several limitations. Firstly, the national administrative inpatient data used does not include the time of admission, which has limited our ability to measure the weekend more precisely than midnight on Friday to midnight on Sunday, although this is common to most of the evidence on weekend effects based on national settings. [1, 2, 18, 20–24, 27, 29, 31, 42] The inpatient data lacks information about disease duration, severity and treatment, while the principal diagnoses are not always accurate. [55] The coding of patient co-morbidities, although established through record linkage to previous admissions during the preceding five study years, would still be incomplete in some cases. The classification of study disorders was also constrained in some cases by the lack of granularity in ICD coding, for example for distinguishing different forms of non-infective gastroenteritis, injuries, skin infections and diabetes mellitus, etc. Despite these limitations, the sizes of the populations studied – and the strongly concordant findings across the two services – suggest external and internal validity.

Whilst we adjusted for patient co-morbidities, national administrative inpatient data does not allow adequate adjustment for disease severity. A recent study of acute admissions to three Oxford hospitals reported that 33% of excess mortality for admissions on Saturdays and 52% on Sundays could be explained through differences in biochemistry and haematology tests. [28] Further research is required, based on linkage of hospital discharge data with laboratory results that measure disease severity and/or degree of organ failure, preferably in unselected national or large regional settings.

## Conclusions

This study provides further insight into how acute disorders may be susceptible to weekend mortality effects. It shows that high mortality on the day of admission and changes in the source of admission at weekends offer some explanation for weekend effects across the study disorders. However, the key explanatory variables are reductions in admission rates at weekends and the medical disease category, which together explained more than 60% of all variation in weekend mortality effects.

We found that patients admitted on weekends overall were slightly younger, had slightly shorter lengths of stay and slightly lower numbers of recoded co-morbidities, than those admitted on week days, which does not suggest that patients admitted at weekend overall are more sick. It may also reflect that avoidable admissions from Emergency Departments at weekends in some hospitals may result from a lack of community and home care services, senior consultant cover and diagnostic services, so that some milder cases are admitted at weekends. [56, 57]

Nonetheless, for conditions with the largest weekend effects, reductions in admission rates at weekend were mostly very large (> 40%). Large reductions in admission rates at weekends could indicate reductions in out of hours service provision on weekends. A reduction in service provision could lead to both a lower admission rate and a higher admission threshold, [29, 58] whereby more severe cases are admitted more often than less severe cases. We found that admission rates were highest on Mondays and mortality was also higher on Mondays than on all other normal week days, which indicates that some possibly deferred admissions may have become more severe by Monday. For certain types of medical conditions such as cancers, weekend availability of services in the community (such as hospice care) or in hospital (specialist therapy) may also affect admission patterns and lead to case mix differences between week days and weekends.

Trauma is a notable exception with increases in admissions at weekends. There is a general acceptance that seriously injured trauma patients need to be seen on the day of the incident, so trauma services are configured to work on a 24/7 basis. The increases in weekend admission rates for trauma and reductions in admission rates for most medical conditions, with no weekend mortality effect for trauma but large consistent effects for 14 of the medical conditions is further evidence of case mix differences in weekend medical admissions. This is supported by the lack of weekend admission and mortality effects for conditions with specialist centres/units that maintain services at weekends.

To summarise, this is one of the largest population studies that provides further evidence as to which acute disorders, across an extensive range of conditions, are susceptible to weekend mortality effects, and possible explanations for this. It shows that large weekend effects are mostly confined to certain types of medical condition. These are largely associated with greatly reduced admission rates at weekends.

### Additional file

Additional file 1: **Table S1.** Patients admitted on weekends and on week days for major acute disorders across England and Wales, compared according to source of admission and social deprivation (DOCX 74 kb)

## Data Availability

The national inpatient and mortality data used in this study is publicly available to other researchers from NHS Digital (English data) and from the NHS Wales Informatics Service (Welsh data). Data sharing is subject to approval by these data custodians for use by named researchers.

## Appendix

**Table 4:** The 66 study disorders with corresponding ICD-10 codes

Circulatory disorders:	
Acute myocardial infarction	I21
Stroke	I61-I64
Subarachnoid haemorrhage	I60
Heart failure	I50
Angina	I20
Valvular heart disease	I05-I08, I34-I37, Q22, Q23
Pulmonary embolism	I26
Atrial fibrillation	I48
Abdominal aortic aneurysm	I71
Peripheral vascular disease	I73
Arterial embolism & thrombosis	I74
Phlebitis & thombophlebitis	I80
Hypertension	I11-I15
Respiratory disorders:	
COPD	J40-J44
Pulmonary oedema	J81
Pneumonia	J12-J18
Pneumonitis	J69
Lower respiratory infections	J20-J22
Pleural effusion	J90, J91
Other interstitial respiratory disease	J84
Respiratory failure	J96
Gastrointestinal disorders:	
Upper gastrointestinal bleeding	I85.0, K22.6, K22.8, K25.0, K25.2, K25.4, K25.6, K26.0, K26.2, K26.4, K26.6, K27.0, K27.2, K27.4, K27.6, K28.0, K28.2, K28.4, K28.6, K29.0, K92.0, K92.1, K92.2^a^
Perforated peptic ulcer & peritonitis	K25.1, K25.2, K25.5, K25.6, K26.1, K26.2, K26.5, K26.6, K27.1, K27.2, K27.5, K27.6, K65
Diverticular disease	K57.2-K57.9
Intestinal obstruction	K56
Herniae	K40-K43, K45, K46
Alcoholic liver disease	K70
Other liver disease	K71-K77
Gallstone disease	K80, K81
Acute pancreatitis	K85
Intestinal infections	A00-A09
Noninfective gastroenteritis	K52
Constipation	K59
Trauma:	
Hip fracture	S72.0, S72.1, S72.2 and in people aged 65+ years, S72.9
Traumatic brain injury	S06
Other head injury	S01-S05, S07-S09
Shoulder & upper arm injury	S40-S49
Thoracic & abdominal injury	S20-S31, S35, S36, S38, S39
Fracture of lumbar spine & pelvis	S32
Drug poisoning	T36-T50
Cancers:	
Oesophageal	C15
Gastric	C16
Colorectal	C18-C20
Pancreatic	C25
Liver	C22
Breast	C50
Lung	C33, C34
Prostate	C61
Ovarian	C56
Lymphomas	C81-C96
Bladder	C67
Kidney	C64
Brain	C71
Ageing-related disorders:	
Alzheimer’s disease	G30
Dementia	F00-F03, F05.1
Age-related physical debility	R54
Other disorders:	
Acute renal failure	N17
Urinary tract infections	N10-N12, N15.0, N30, N34, N36, N39.0
Diabetes	E10-E14
Septicaemia	A40, A41
Skin infections	L00-L09
Anaemias	D50-D64
Pressure lower limb ulcers	L89, L97
Disorientation - unspecified	R41.0
Malaise and fatigue	R53
Syncope and collapse	R55

^a^Unless present with a diagnosis of lower gastrointestinal disease (C17.1-C21, K50-K52, K55-K57 or K60-K63)

**Table 5:** Details of the exposure measures and quintile categories used in the multivariable regression analysis

Reductions in admission rates at weekends:	Measured as percentage reductions in admission rates at weekends compared with week days
Medical disease category:	Circulatory, respiratory, gastrointestinal, trauma, cancers, ageing-related and other disorders
Mortality on the day of admission:	Measured as percentages of deaths on the day of admission compared with all deaths by day 30
Changes in the source of admission at weekends:	Measured as the risk ratio at weekends (compared with week days) of admissions through hospital emergency departments rather than via primary care or consultant clinics
Mean patient age:	Measured in age (years)
Patient social deprivation:	Measured as the percentage of patients admitted with the most deprived social deprivation groups IV and V

## Abbreviations

COPD: Chronic obstructive pulmonary disease
ICD: International Classification of Diseases
NHS: National Health Service
UK: United Kingdom
