# Supplementary material for: Weekend admissions and mortality for major acute disorders across England and Wales: record linkage cohort studies
Source: BMC Health Serv Res. 2019 Sep 2;19:619. doi: 10.1186/s12913-019-4286-8 (PMC6720086; doi:10.1186/s12913-019-4286-8)
Supplement: Supplementary file 1 — Table S1. Patients admitted on weekends and on week days for major acute disorders across England and Wales, compared according to source of admission and social deprivation (DOCX 74 kb) [file 12913_2019_4286_MOESM1_ESM.docx]

**Supplementary Online Table Patients admitted on weekends and on week days for major acute disorders across England and Wales, compared according to source of admission and social deprivation**

|  | **England** | | | | | | |  | **Wales** | | | | | | |
| --- | --- | --- | --- | --- | --- | --- | --- | --- | --- | --- | --- | --- | --- | --- | --- |
|  | **Source of admission** | | | | |  | **Social deprivation** |  | **Source of admission** | | | | |  | **Social deprivation** |
|  | Emergency  Department (%) |  | Primary  care (%) |  | Consultant  clinic (%) |  | Quintiles IV  and V (%) |  | Emergency  Department (%) |  | Primary  care (%) |  | Consultant  clinic (%) |  | Quintiles IV  and V (%) |
|  | Week Week  Day end |  | Week Week  day end |  | Week Week  day end |  | Week Week  day end |  | Week Week  day end |  | Week Week  Day end |  | Week Week  day end |  | Week Week  day end |

| **Circulatory diseases:** |  |  |  |  |  |  |  |  |  |  |  |  |  |  |  |  |  |  |  |  |  |  | |  |
| --- | --- | --- | --- | --- | --- | --- | --- | --- | --- | --- | --- | --- | --- | --- | --- | --- | --- | --- | --- | --- | --- | --- | --- | --- |
| Acute myocardial infarction | 74.7 | 81.0 |  | 11.0 | 4.8 |  | 0.9 | 0.1 |  | 41.7 | 42.1 |  | 67.9 | 79.6 |  | 22.9 | 10.4 |  | 0.5 | 0.1 |  | 42.8 | 43.1 | |
| Stroke | 77.6 | 86.3 |  | 16.1 | 9.0 |  | 0.9 | 0.1 |  | 41.2 | 41.0 |  | 64.3 | 80.0 |  | 28.6 | 16.3 |  | 0.7 | 0.1 |  | 42.0 | 42.1 | |
| Subarachnoid haemorrhage | 69.8 | 75.8 |  | 10.3 | 5.7 |  | 0.8 | 0.3 |  | 42.3 | 43.7 |  | 72.9 | 83.2 |  | 16.3 | 9.8 |  | 0.5 | 0.0 |  | 44.5 | 42.6 | |
| Heart failure | 61.6 | 82.4 |  | 28.5 | 13.0 |  | 3.3 | 0.2 |  | 44.6 | 45.1 |  | 45.2 | 70.7 |  | 47.3 | 24.9 |  | 3.2 | 0.1 |  | 45.1 | 45.5 | |
| Angina | 80.3 | 90.8 |  | 12.4 | 3.6 |  | 1.5 | 0.1 |  | 47.3 | 47.6 |  | 65.3 | 83.3 |  | 27.8 | 9.6 |  | 1.4 | 0.1 |  | 47.0 | 47.4 | |
| Valvular heart disease | 60.9 | 81.2 |  | 19.9 | 9.1 |  | 7.0 | 0.9 |  | 38.1 | 39.3 |  | 50.7 | 74.8 |  | 33.2 | 17.5 |  | 6.0 | 0.2 |  | 38.1 | 39.6 | |
| Pulmonary embolism | 63.4 | 82.7 |  | 26.8 | 12.5 |  | 3.2 | 0.3 |  | 39.3 | 41.0 |  | 49.8 | 71.0 |  | 42.2 | 24.1 |  | 3.1 | 0.3 |  | 41.1 | 43.4 | |
| Atrial fibrillation | 67.4 | 85.6 |  | 23.2 | 9.4 |  | 2.8 | 0.2 |  | 37.3 | 36.8 |  | 50.0 | 77.1 |  | 42.6 | 18.6 |  | 3.0 | 0.1 |  | 38.3 | 37.6 | |
| Abdominal aortic aneurysm | 71.8 | 83.5 |  | 15.6 | 9.2 |  | 4.9 | 0.6 |  | 40.2 | 40.6 |  | 59.3 | 73.0 |  | 25.7 | 20.0 |  | 7.1 | 0.9 |  | 40.9 | 45.7 | |
| Peripheral vascular disease | 43.2 | 68.7 |  | 34.0 | 21.9 |  | 11.9 | 1.8 |  | 48.8 | 49.8 |  | 24.3 | 57.7 |  | 54.3 | 36.8 |  | 11.2 | 1.2 |  | 47.1 | 47.7 | |
| Arterial embolism & thrombosis | 50.7 | 72.7 |  | 27.0 | 16.4 |  | 10.9 | 1.0 |  | 47.9 | 46.8 |  | 40.4 | 65.5 |  | 40.6 | 28.3 |  | 10.5 | 0.9 |  | 48.7 | 51.4 | |
| Phlebitis & thrombophlebitis | 39.4 | 63.0 |  | 48.3 | 28.9 |  | 4.6 | 0.5 |  | 43.1 | 47.8 |  | 34.7 | 48.9 |  | 51.9 | 45.7 |  | 8.6 | 0.7 |  | 46.7 | 46.2 | |
| Hypertension | 49.0 | 70.0 |  | 27.5 | 14.4 |  | 6.2 | 1.7 |  | 48.5 | 50.9 |  | 30.7 | 56.6 |  | 51.4 | 30.6 |  | 7.8 | 1.4 |  | 44.5 | 45.0 | |
|  |  |  |  |  |  |  |  |  |  |  |  |  |  |  |  |  |  |  |  |  |  |  |  | |
| **Respiratory diseases:** |  |  |  |  |  |  |  |  |  |  |  |  |  |  |  |  |  |  |  |  |  |  |  | |
| COPD | 70.0 | 84.6 |  | 22.9 | 10.7 |  | 1.2 | 0.1 |  | 57.7 | 58.8 |  | 52.6 | 74.1 |  | 42.5 | 21.5 |  | 1.3 | 0.1 |  | 57.4 | 58.5 | |
| Pulmonary oedema | 74.1 | 86.0 |  | 16.9 | 8.3 |  | 3.1 | 0.3 |  | 48.1 | 47.9 |  | 65.0 | 81.4 |  | 29.1 | 14.2 |  | 1.8 | 0.3 |  | 46.4 | 43.5 | |
| Pneumonia | 70.9 | 81.8 |  | 23.1 | 14.0 |  | 1.0 | 0.1 |  | 46.5 | 46.9 |  | 52.5 | 68.6 |  | 42.1 | 27.1 |  | 1.0 | 0.1 |  | 47.0 | 46.8 | |
| Pneumonitis | 76.0 | 83.6 |  | 18.8 | 12.5 |  | 0.6 | 0.1 |  | 45.1 | 45.5 |  | 55.4 | 66.1 |  | 38.5 | 28.1 |  | 0.6 | 0.0 |  | 44.2 | 43.1 | |
| Lower respiratory infections | 64.6 | 78.1 |  | 27.9 | 16.3 |  | 1.3 | 0.1 |  | 47.0 | 47.4 |  | 44.9 | 63.6 |  | 49.8 | 31.7 |  | 1.2 | 0.2 |  | 46.4 | 47.0 | |
| Pleural effusion | 50.1 | 77.3 |  | 28.7 | 15.3 |  | 9.4 | 0.6 |  | 39.9 | 44.1 |  | 34.4 | 62.4 |  | 47.2 | 30.7 |  | 10.8 | 0.8 |  | 44.4 | 47.0 | |
| Other interstitial respiratory disease | 55.0 | 79.8 |  | 28.4 | 13.5 |  | 5.8 | 0.2 |  | 42.2 | 43.2 |  | 42.4 | 63.6 |  | 48.4 | 31.0 |  | 3.9 | 0.2 |  | 45.7 | 49.3 | |
| Respiratory failure | 74.3 | 87.0 |  | 16.6 | 9.1 |  | 2.5 | 0.3 |  | 52.9 | 52.7 |  | 53.3 | 73.2 |  | 38.0 | 21.8 |  | 2.8 | 0.2 |  | 51.8 | 49.7 | |
|  |  |  |  |  |  |  |  |  |  |  |  |  |  |  |  |  |  |  |  |  |  |  |  | |
| **Gastrointestinal disorders:** |  |  |  |  |  |  |  |  |  |  |  |  |  |  |  |  |  |  |  |  |  |  |  | |
| Upper gastrointestinal bleeding | 68.8 | 83.7 |  | 25.5 | 12.9 |  | 0.8 | 0.1 |  | 48.8 | 50.5 |  | 52.2 | 72.5 |  | 44.6 | 25.5 |  | 0.8 | 0.0 |  | 47.7 | 50.2 | |
| Perforated peptic ulcer & peritonitis | 68.5 | 78.4 |  | 19.4 | 12.9 |  | 3.7 | 0.7 |  | 48.4 | 49.8 |  | 58.0 | 69.6 |  | 32.6 | 25.6 |  | 3.9 | 2.0 |  | 48.8 | 48.3 | |
| Diverticular disease | 59.7 | 74.0 |  | 33.4 | 21.6 |  | 1.5 | 0.2 |  | 39.2 | 40.6 |  | 44.0 | 59.1 |  | 42.9 | 33.0 |  | 1.0 | 0.1 |  | 41.5 | 40.5 | |
| Intestinal obstruction | 62.8 | 72.4 |  | 29.4 | 22.2 |  | 2.0 | 0.2 |  | 38.6 | 39.2 |  | 47.3 | 56.5 |  | 47.0 | 41.1 |  | 2.4 | 0.2 |  | 41.7 | 40.2 | |
| Herniae | 55.7 | 70.6 |  | 36.9 | 24.2 |  | 1.3 | 0.2 |  | 44.0 | 45.0 |  | 42.6 | 58.3 |  | 54.1 | 40.1 |  | 1.1 | 0.1 |  | 45.5 | 46.0 | |
| Alcoholic liver disease | 60.7 | 82.7 |  | 28.0 | 12.1 |  | 3.4 | 0.4 |  | 60.1 | 62.2 |  | 44.5 | 73.6 |  | 49.2 | 24.4 |  | 3.3 | 0.1 |  | 60.6 | 61.6 | |
| Other liver disease | 54.1 | 75.5 |  | 27.5 | 15.8 |  | 6.2 | 0.5 |  | 48.2 | 50.6 |  | 37.2 | 66.8 |  | 49.7 | 30.4 |  | 7.6 | 0.4 |  | 45.8 | 46.0 | |
| Gallstone disease | 67.4 | 78.5 |  | 26.1 | 17.3 |  | 1.3 | 0.1 |  | 45.7 | 46.4 |  | 55.0 | 66.7 |  | 42.1 | 31.8 |  | 0.8 | 0.1 |  | 45.7 | 45.5 | |
| Acute pancreatitis | 78.2 | 85.2 |  | 17.4 | 11.6 |  | 0.7 | 0.1 |  | 50.3 | 50.5 |  | 65.1 | 76.8 |  | 32.9 | 22.0 |  | 0.5 | 0.1 |  | 49.2 | 48.3 | |
| Intestinal infections | 66.1 | 75.4 |  | 26.6 | 18.8 |  | 1.3 | 0.2 |  | 46.2 | 46.0 |  | 43.9 | 55.2 |  | 50.6 | 40.9 |  | 1.4 | 0.1 |  | 44.0 | 44.3 | |
| Noninfective gastroenteritis | 63.6 | 75.2 |  | 27.5 | 18.1 |  | 1.9 | 0.2 |  | 47.6 | 47.9 |  | 43.2 | 57.3 |  | 51.4 | 37.3 |  | 1.6 | 0.3 |  | 44.8 | 46.9 | |
| Constipation | 59.7 | 73.3 |  | 32.1 | 20.5 |  | 1.5 | 0.2 |  | 47.9 | 48.8 |  | 41.0 | 54.5 |  | 55.7 | 43.3 |  | 1.3 | 0.2 |  | 48.6 | 48.2 | |

**Supplementary Online Table cont'd**

|  | **England** | | | | | | |  | **Wales** | | | | | | |
| --- | --- | --- | --- | --- | --- | --- | --- | --- | --- | --- | --- | --- | --- | --- | --- |
|  | **Source of admission** | | | | |  | **Social deprivation** |  | **Source of admission** | | | | |  | **Social deprivation** |
|  | Emergency  Department (%) |  | Primary  care (%) |  | Consultant  clinic (%) |  | Quintiles IV  and V (%) |  | Emergency  Department (%) |  | Primary  care (%) |  | Consultant  clinic (%) |  | Quintiles IV  and V (%) |
|  | Week Week  day end |  | Week Week  day end |  | Week Week  day end |  | Week Week  Day end |  | Week Week  day end |  | Week Week  day end |  | Week Week  day end |  | Week Week  day end |

| **Trauma:** |  |  |  |  |  |  |  |  |  |  |  |  |  |  |  |  |  |  |  |  | | |  |  |  |
| --- | --- | --- | --- | --- | --- | --- | --- | --- | --- | --- | --- | --- | --- | --- | --- | --- | --- | --- | --- | --- | --- | --- | --- | --- | --- |
| Hip fracture | 96.2 | 97.5 |  | 1.8 | 1.0 |  | 0.4 | 0.1 |  | 38.3 | 38.6 |  | 91.0 | 97.1 |  | 3.6 | 1.6 |  | 0.4 | 0.0 |  | 39.9 | | 41.7 |  |
| Traumatic brain injury | 86.1 | 90.0 |  | 5.0 | 1.7 |  | 0.4 | 0.1 |  | 44.3 | 46.2 |  | 82.2 | 92.9 |  | 8.9 | 3.8 |  | 0.6 | 0.0 |  | 44.2 | | 47.2 |  |
| Other head injury | 91.6 | 95.1 |  | 2.1 | 1.0 |  | 2.7 | 0.5 |  | 51.0 | 52.7 |  | 89.6 | 97.0 |  | 4.0 | 1.6 |  | 4.4 | 0.2 |  | 48.8 | | 50.4 |  |
| Shoulder & upper arm injury | 86.8 | 95.4 |  | 2.2 | 1.2 |  | 7.2 | 1.3 |  | 42.4 | 42.8 |  | 78.1 | 95.6 |  | 4.2 | 2.4 |  | 14.0 | 0.7 |  | 41.2 | | 43.4 |  |
| Thoracic & abdominal injury | 90.0 | 95.0 |  | 6.8 | 2.6 |  | 0.6 | 0.1 |  | 46.5 | 48.3 |  | 85.8 | 94.1 |  | 11.6 | 5.0 |  | 0.7 | 0.1 |  | 45.8 | | 46.8 |  |
| Fracture of lumbar spine & pelvis | 92.3 | 95.8 |  | 4.3 | 2.0 |  | 0.8 | 0.1 |  | 38.5 | 37.9 |  | 87.3 | 94.4 |  | 6.9 | 4.2 |  | 1.4 | 0.1 |  | 41.0 | | 43.7 |  |
| Drug poisoning | 96.0 | 97.4 |  | 2.4 | 1.3 |  | 0.1 | 0.0 |  | 59.6 | 60.1 |  | 80.7 | 82.9 |  | 6.7 | 4.5 |  | 0.1 | 0.0 |  | 60.2 | | 61.2 |  |
|  |  |  |  |  |  |  |  |  |  |  |  |  |  |  |  |  |  |  |  |  |  |  | |  |  |
| **Cancers:** |  |  |  |  |  |  |  |  |  |  |  |  |  |  |  |  |  |  |  |  |  |  | |  |  |
| Oesophageal | 37.8 | 59.4 |  | 29.9 | 22.9 |  | 12.2 | 1.3 |  | 41.0 | 41.4 |  | 27.3 | 49.5 |  | 49.7 | 40.3 |  | 10.9 | 1.5 |  | 42.2 | | 40.2 |  |
| Gastric | 41.9 | 62.6 |  | 30.1 | 20.2 |  | 10.4 | 1.3 |  | 45.7 | 45.9 |  | 29.3 | 52.8 |  | 47.8 | 43.6 |  | 7.4 | 0.7 |  | 44.4 | | 47.3 |  |
| Colorectal | 42.7 | 61.2 |  | 30.5 | 22.2 |  | 10.5 | 1.4 |  | 39.0 | 39.3 |  | 30.8 | 48.4 |  | 52.5 | 37.7 |  | 7.6 | 0.7 |  | 39.2 | | 41.3 |  |
| Pancreatic | 39.9 | 59.7 |  | 34.1 | 24.6 |  | 10.6 | 1.4 |  | 37.8 | 40.3 |  | 27.2 | 49.5 |  | 55.6 | 45.3 |  | 7.8 | 0.5 |  | 40.1 | | 43.5 |  |
| Liver | 42.4 | 64.0 |  | 29.4 | 19.0 |  | 10.4 | 1.9 |  | 44.5 | 46.8 |  | 30.3 | 50.0 |  | 53.0 | 40.7 |  | 8.6 | 1.1 |  | 45.9 | | 45.2 |  |
| Breast | 31.0 | 46.1 |  | 20.0 | 16.8 |  | 18.9 | 3.8 |  | 40.5 | 41.7 |  | 26.1 | 45.9 |  | 42.5 | 37.6 |  | 10.5 | 1.1 |  | 36.7 | | 39.3 |  |
| Lung | 47.0 | 69.8 |  | 26.7 | 17.2 |  | 10.6 | 0.9 |  | 49.6 | 50.6 |  | 34.1 | 58.9 |  | 45.6 | 34.5 |  | 8.8 | 0.3 |  | 50.1 | | 51.1 |  |
| Prostate | 41.5 | 62.1 |  | 33.3 | 23.7 |  | 11.0 | 1.4 |  | 35.8 | 36.6 |  | 26.4 | 54.4 |  | 52.6 | 39.5 |  | 8.7 | 0.7 |  | 33.7 | | 33.8 |  |
| Ovarian | 30.2 | 49.1 |  | 26.7 | 20.6 |  | 17.6 | 3.0 |  | 35.9 | 37.0 |  | 21.5 | 45.2 |  | 50.2 | 44.2 |  | 10.2 | 1.0 |  | 37.1 | | 39.7 |  |
| Lymphomas | 26.9 | 45.7 |  | 18.9 | 14.9 |  | 21.2 | 3.4 |  | 36.6 | 37.0 |  | 22.2 | 39.5 |  | 33.6 | 28.2 |  | 23.8 | 1.1 |  | 37.1 | | 38.1 |  |
| Bladder | 45.5 | 65.3 |  | 28.9 | 21.0 |  | 10.7 | 1.4 |  | 40.6 | 40.6 |  | 31.5 | 59.2 |  | 49.3 | 36.6 |  | 8.3 | 0.7 |  | 40.8 | | 43.8 |  |
| Kidney | 38.6 | 59.7 |  | 26.3 | 19.2 |  | 14.4 | 1.9 |  | 39.7 | 41.8 |  | 29.1 | 50.5 |  | 51.6 | 45.2 |  | 7.3 | 0.5 |  | 39.6 | | 34.9 |  |
| Brain | 49.3 | 65.8 |  | 23.7 | 15.5 |  | 8.1 | 1.2 |  | 34.7 | 35.1 |  | 39.2 | 58.3 |  | 39.2 | 27.4 |  | 4.8 | 0.6 |  | 37.4 | | 36.9 |  |
|  |  |  |  |  |  |  |  |  |  |  |  |  |  |  |  |  |  |  |  |  |  |  | |  |  |
| **Ageing-related disorders:** |  |  |  |  |  |  |  |  |  |  |  |  |  |  |  |  |  |  |  |  |  |  | |  |  |
| Alzheimer's disease | 39.7 | 69.0 |  | 21.0 | 15.4 |  | 3.9 | 0.9 |  | 41.4 | 42.3 |  | 21.0 | 49.4 |  | 30.6 | 31.0 |  | 1.6 | 0.3 |  | 41.3 | | 41.7 |  |
| Dementia | 45.7 | 71.6 |  | 24.6 | 17.2 |  | 3.8 | 0.7 |  | 43.4 | 43.8 |  | 23.0 | 53.8 |  | 32.8 | 29.1 |  | 2.9 | 0.7 |  | 41.2 | | 44.9 |  |
| Age-related physical debility | 77.1 | 88.1 |  | 18.9 | 9.2 |  | 0.4 | 0.1 |  | 42.8 | 43.3 |  | 63.3 | 81.4 |  | 29.9 | 14.7 |  | 1.1 | 0.0 |  | 45.0 | | 45.9 |  |
|  |  |  |  |  |  |  |  |  |  |  |  |  |  |  |  |  |  |  |  |  |  |  | |  |  |
| **Other acute disorders:** |  |  |  |  |  |  |  |  |  |  |  |  |  |  |  |  |  |  |  |  |  |  | |  |  |
| Urinary tract infections | 69.3 | 79.6 |  | 24.7 | 15.7 |  | 0.9 | 0.2 |  | 45.3 | 45.3 |  | 48.3 | 63.0 |  | 47.3 | 33.7 |  | 0.9 | 0.1 |  | 46.1 | | 49.0 |  |
| Acute renal failure | 57.4 | 77.3 |  | 31.9 | 16.6 |  | 2.7 | 0.5 |  | 44.0 | 46.2 |  | 38.8 | 64.3 |  | 53.2 | 31.2 |  | 3.2 | 0.5 |  | 44.7 | | 47.2 |  |
| Diabetes | 60.4 | 81.0 |  | 26.0 | 14.2 |  | 7.3 | 0.4 |  | 53.4 | 55.8 |  | 42.6 | 65.9 |  | 46.1 | 29.7 |  | 7.1 | 0.5 |  | 50.8 | | 49.9 |  |
| Septicaemia | 66.9 | 75.2 |  | 21.0 | 15.3 |  | 2.8 | 0.5 |  | 42.6 | 42.6 |  | 50.4 | 61.6 |  | 37.7 | 28.4 |  | 2.9 | 0.7 |  | 41.6 | | 43.0 |  |
| Skin infections | 57.5 | 75.9 |  | 33.0 | 18.3 |  | 3.2 | 0.5 |  | 49.0 | 49.7 |  | 43.7 | 64.4 |  | 51.0 | 33.1 |  | 3.2 | 0.3 |  | 48.8 | | 47.7 |  |
| Anaemias | 43.6 | 73.8 |  | 37.4 | 15.9 |  | 5.9 | 1.3 |  | 48.1 | 53.8 |  | 24.9 | 54.3 |  | 62.6 | 37.5 |  | 7.1 | 1.0 |  | 44.7 | | 47.3 |  |
| Pressure lower limb ulcers | 46.3 | 73.5 |  | 34.0 | 19.3 |  | 11.0 | 1.2 |  | 47.7 | 49.5 |  | 26.3 | 50.3 |  | 58.2 | 43.7 |  | 9.6 | 2.0 |  | 46.1 | | 49.0 |  |
| Disorientation - unspecified | 66.8 | 79.4 |  | 26.7 | 16.1 |  | 0.7 | 0.1 |  | 44.1 | 44.7 |  | 45.8 | 62.5 |  | 49.0 | 34.0 |  | 0.7 | 0.2 |  | 44.3 | | 46.4 |  |
| Malaise & fatigue | 61.7 | 79.9 |  | 27.4 | 13.7 |  | 2.0 | 0.2 |  | 48.3 | 49.5 |  | 35.3 | 56.4 |  | 54.8 | 35.6 |  | 2.0 | 0.3 |  | 48.0 | | 45.1 |  |
| Syncope & collapse | 88.0 | 94.0 |  | 8.1 | 3.7 |  | 0.9 | 0.1 |  | 44.6 | 43.6 |  | 73.8 | 84.0 |  | 19.0 | 10.0 |  | 1.1 | 0.0 |  | 44.0 | | 43.3 |  |
